# Evaluation of the frequency of non-motor symptoms of Parkinson’s disease in adult patients with Gaucher disease type 1

**DOI:** 10.1186/s13023-019-1079-4

**Published:** 2019-05-10

**Authors:** Matheus V. M. B. Wilke, Alícia D. Dornelles, Artur S. Schuh, Filippo P. Vairo, Suelen P. Basgalupp, Marina Siebert, Tatiele Nalin, Otavio B. Piltcher, Ida V. D. Schwartz

**Affiliations:** 1 Postgraduate Program in Medical Sciences, Faculdade de Medicina, Universidade Federal do Rio Grande do Sul, Porto Alegre, RS Brazil; 2 Medical Genetics Service, Hospital de Clínicas de Porto Alegre, Porto Alegre, RS Brazil; 3 Department of Pharmacology, Universidade Federal do Rio Grande do Sul, Porto Alegre, Brazil; 4 Center for Individualized Medicine, Mayo Clinic, Rochester, MN USA; 5 Department of Clinical Genomics, Mayo Clinic, Rochester, MN USA; 6 Graduate Program in Sciences of Gastroenterology and Hepatology, Universidade Federal do Rio Grande do Sul (UFRGS), Porto Alegre, RS Brazil; 7 Laboratory Research Unit, Experimental Research Center, Hospital de Clínicas de Porto Alegre (HCPA), Porto Alegre, RS Brazil; 8 Department of Ophthalmology and Otorhinolaryngology, Hospital de Clínicas de Porto Alegre, Porto Alegre, RS Brazil; 9 Department of Genetics, Universidade Federal do Rio Grande do Sul, Porto Alegre, Brazil; 10 BRAIN Laboratory, Hospital de Clínicas de Porto Alegre, Porto Alegre, RS Brazil

**Keywords:** Gaucher disease, Parkinson’s disease, Non-motor symptoms

## Abstract

**Background:**

Gaucher disease (GD) is caused by deficiency of beta-glucocerebrosidase (GCase) due to biallelic variations in the *GBA1* gene. Parkinson’s disease (PD) is the second most common neurodegenerative condition. The classic motor symptoms of PD may be preceded by many non-motor symptoms (NMS), which include hyposmia, rapid eye movement (REM) sleep behavior disorder, constipation, cognitive impairment, and depression. Population studies have identified mutations in *GBA1* as the main risk factor for idiopathic PD. The present study sought to evaluate the prevalence of NMS in a cohort of patients with GD type 1 from Southern Brazil.

**Methodology:**

This is an observational, cross-sectional study, with a convenience sampling strategy. Cognition was evaluated by the Montreal Cognitive assessment (MoCa), daytime sleepiness by the Epworth Scale, depression by the Beck Inventory, constipation by the Unified Multiple System Atrophy Rating Scale, and REM sleep behavior disorder by the Single-Question Screen; hyposmia by the Sniffin’ Sticks. Motor symptoms were assessed with part III of the Unified Parkinson’s Disease Rating Scale. All patients were also genotyped for the *GBA1* 3′-UTR SNP (rs708606).

**Results:**

Twenty-three patients (female = 13; on enzyme replacement therapy = 21, substrate reduction therapy = 2) with a mean age of 41.45 ± 15.3 years (range, 22–67) were included. Eight patients were found to be heterozygous for the 3′-UTR SNP (rs708606). Fourteen patients (8 over age 40 years) presented at least one NMS; daytime sleepiness was the most frequent (*n* = 10). Two patients (aged 63 and 64, respectively) also presented motor symptoms, probably drug-related.

**Conclusions:**

NMS were prevalent in this cohort. We highlight the importance of a multidisciplinary follow-up focusing on earlier diagnosis of PD, especially for patients with GD type 1 over the age of 40.

## Introduction

Gaucher disease (GD, OMIM 230800) is caused by deficient activity of beta-glucocerebrosidase (GCase) due to biallelic pathogenic variants in the *GBA1* gene located at chromosome 1q21. GD is one of the most common lysosomal disorders, with an estimated worldwide incidence of 1 case per 57,000 live births [1, 2]. Three clinical forms of GD are conventionally classified based on the neurological involvement. Type 1 is considered non-neuronopathic, whereas types 2 and 3 are considered the neuronopathic forms [3]. More than 400 mutations in the *GBA1* have been described, with c.1226A > G (N370S) being the most frequent in the GD type 1 population [4].

Parkinson’s disease (PD) is the second most common neurodegenerative condition, affecting 2% of the population over age 60 years and 4% of the population over age 80 [5]. The motor symptoms of PD are preceded by a prodromal period of up to 20 years. The so-called non-motor symptoms (NMS) that occur during this prodrome, such as hyposmia, rapid eye movement (REM) sleep disorder, daytime drowsiness, constipation, depression, and anxiety, may represent the beginning of the pathological process of PD [6–8].

Population studies have identified *GBA1* mutations as the main risk factor for idiopathic PD (iPD). Carriers for mutations in *GBA1* and patients with GD have a lifetime relative risk of developing PD greater than that of the overall population, which depends on the age (for instance, the penetrance of PD in heterozygous carriers of GtBA1 mutations is estimated at 13.7% at the age of 60 and 29.7% at the age of 80) and on the the mutations (the odds ratios for PD in *GBA1* mutation heterozygous ranged between 2.84 and 21.29 depending on the severity of the mutation) [9–12]. A small cohort study also suggested that not only mutations in exonic regions but also a single nucleotide polymorphism (SNP) in the 3′-UTR of *GBA1* (rs708606) in the intron-exon boundaries is implicated in the cognitive symptoms of PD [13].

Within this context, our main objective was to evaluate the prevalence of NMS of PD in a cohort of Brazilian patients with GD type 1.

## Materials and methods

**Fig. 1:**
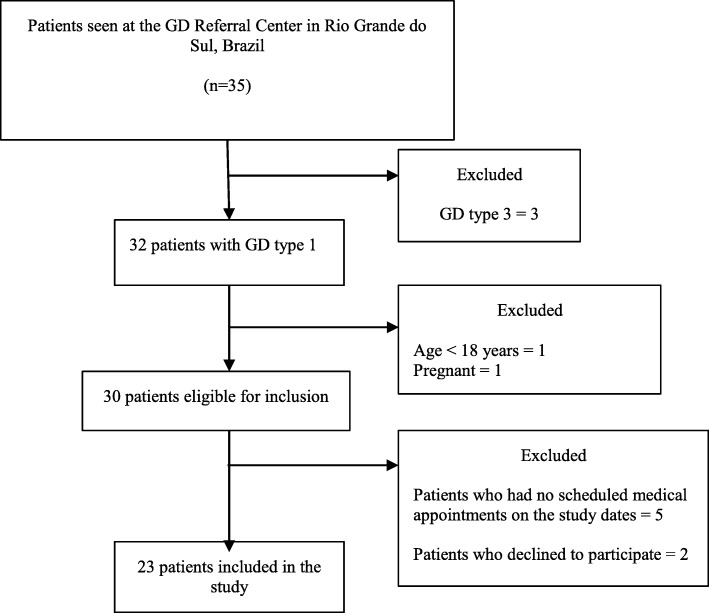
Flow diagram of patient enrollment

This is an observational, cross-sectional study. All patients with GD type 1 seen at the Reference Center for GD in Rio Grande do Sul, Brazil, were invited to participate during their routine follow-up visits from March to August 2018. Patients were required to meet the following inclusion criteria: a) GD diagnosis confirmed by low GCase activity in leukocytes or fibroblasts and/or genetic analysis; and b) age 18 years or older. The exclusion criteria were: a) history of parkinsonian manifestations, as previously reported in medical records; b) known diagnosis of PD; and c) pregnancy. Figure 1 shows a flow diagram of patient enrollment.

Patients who agreed to participate in the study were evaluated by a single doctor (MW) who collected clinical data, such as family history of PD and the presence of parkinsonian manifestations. Motor symptoms of PD were assessed with part III of the Unified Parkinson’s Disease Rating Scale (MDS-UPDRS III). All patients who scored higher than 0 were referred to a neurologist for further evaluation. Patients were also asked to complete self-report questionnaires to evaluate the following NMS of PD: cognition, daytime sleepiness, depression, constipation, and REM sleep behavior disorder, evaluated respectively by the Montreal Cognitive assessment (MoCa, cutoff for cognitive impairment < 26), the Epworth Sleepiness Scale (ESS, cutoff point > 10), the Beck Depression Inventory (BDI) (cutoff for depression > 14), the subscale of the Unified Multiple System Atrophy Rating Scale (UMSARS, cutoff for constipation ≥2) and the validated Single-Question Screen (RBD-1Q). If the patient answered “yes” to the RBD-1Q question, further investigation with polysomnography (PSG) was offered.

Hyposmia was assessed with the 12-item Sniffin’ Sticks smell identification test (cut off for hyposmia < 9/12). Patients who scored below the cutoff were referred to an ENT doctor to rule out anatomic defects of the airway.

Data on duration of treatment, GD severity, demographic and epidemiological variables, physical and neurologic examination, and laboratory parameters were obtained by a review of medical records. All patients seen at the Reference Center for GD have the *GBA1* gene genotyped through next-generation sequencing (NGS); presence of the 3′-UTR SNP (rs708606; wild form: C, alternative form: T), already described in the literature, was assessed in the Integrative Genomics Viewer (IGV) software.

This study was approved by the local ethics committee.

### Statistical analysis

Numerical variables with normal distribution were expressed as means and standard deviations. For tests where at least 15% of patients had abnormal results, both groups of patients were compared regarding clinical and laboratory findings (hemoglobin, platelet, chitotriosidase, GD severity scores, duration of treatment, current age, age at diagnosis).

The difference between groups was assessed with the Mann–Whitney *U* test for independent variables. The level of statistical significance was set at 5% for all analyses. Statistical calculations were carried out in PASW Statistics for Windows, Version 18.0.

## Results

Twenty-three patients were included in this study (Table 1). The mean age of the sample was 41.4 ± 15.3 years (range, 22–67) with a mean time of treatment duration of 11.5 ± 6.0 years (range, 3–24). The mean age at onset of GD symptoms was 16.7 ± 14.1 years (range, 2–48). A family history of PD was reported by two patients, in second-degree relatives, but these individuals were not available for clinical evaluation. The majority of patients (*n* = 22/23) had at least one N370S allele, with the most common genotype being N370S/Rec*Nci*I (*n* = 10/23). Eight patients were heterozygous for the 3′-UTR SNP in *GBA1* (rs708606); of these, six had at least one NMS of PD. There was no significant association between this finding and clinical or laboratory parameters.

**Table 1:** Demographic and clinical characteristics of patients with Gaucher disease type 1 (*n* = 23)

Patient	Gender	Age (years)	Genotype	*3′-UTR SNP (rs708606) Allele1/Allele2*	*SPX*	*Age at diagnosis (years)*	*Treatment duration (years)*	Current treatment	Severity Scores		Hb (g/dL)	Plat (× 10^9^/L)	Current ChT activity (nmol/hr./mL)
									SSI	DS3			
1	M	22	N370S/G202R	C/C	N	8	14	ERT	0	0	14.7	181	3338
2	F	23	N370S/L444P	C/T	N	20	3	ERT	2	0.7	13.2	191	4943
3	F	23	N370S/Rec*Nci*I	C/C	N	7	15	ERT	0	0	11.8	277	399
4	F	25	N370S/L444P	C/C	N	15	10	ERT	0	0	13.2	152	389
5	M	26	N370S/IVS9 + 1G > A	C/T	N	10	15	ERT	4	1.7	16.2	134	4521
6^a^	F	27	N370S/Rec*Nci*I	C/C	N	10	17	SRT	5	1.6	11.6	157	6314
7	F	28	N370S/L461P + IVS10 + 1G > T	C/C	N	4	24	ERT	1	0.7	12.9	122	3665
8	M	31	N370S/Rec*Nci*I	C/C	N	14	16	ERT	1	2.7	15.1	203	1463
9^a^	F	32	N370S/L444P	C/C	N	11	21	ERT	0	0	13.6	202	2847
10	M	37	N370S/ L444P + E326K	C/T	N	27	6	ERT	1	0	16.0	179	629
11	F	38	N370S/Rec*Nci*I	C/C	N	35	3	ERT	0	0	13.4	235	983
12	F	39	N370S/ L444P + E326K	C/T	N	29	9	SRT	0	0	12.5	167	2242
13	M	41	N370S/L444P	C/T	Y	26	16	ERT	5	1.7	14.2	328	1854
14	M	45	N370S/L444P	C/T	N	37	6	ERT	3	0.5	15.0	205	306
15	F	49	E349K/S366 N	C/T	N	42	6	ERT	5	2.6	15.5	291	616
16	F	51	N370S/Rec*Nci*I	C/C	Y	34	17	ERT	11	2.9	14.2	434	1055
17	M	52	N370S/Rec*Nci*I	C/C	N	44	6	ERT	2	2.2	15.4	120	1451
18	M	57	N370S/Rec*Nci*I	C/C	N	50	7	ERT	3	0	16.4	170	1085
19	F	62	N370S/Rec*Nci*I	C/C	N	42	20	ERT	3	2.5	13.6	149	265
20	M	63	N370S/Rec*Nci*I	C/C	Y	49	11	ERT	9	7	15.3	222	440
21	M	64	N370S/N370S	C/C	N	54	9	ERT	2	1.1	13.3	143	1860
22	F	67	N370S/L444R	C/T	Y	60	7	ERT	4	2.6	14.4	268	240
23	F	67	N370S/Rec*Nci*I	C/C	N	57	6	ERT	4	1.9	14.0	161	223

*3′-UTR SNP (rs708606)* C is the wild form, *SPX* splenectomy, *N* no, *Y* yes, *ERT* enzyme replacement therapy, *SRT* substrate reduction therapy, *SSI* Zimran Severity Score Index (mild = 0–10; moderate = 11–19; severe ≥20), *DS3* Disease Severity Score (mild = < 3.00; moderate = 3.00–5.99; marked = 6.00–19), *Hb* hemoglobin, *Plat* platelet count, *ChT* chitotriosidase activity (normal range: < 78.5 nmol/hr./mL). ^a^Patients with reported family history of Parkinson’s disease

The summary of the findings on the NMS of PD is provided in Table 2. Nine patients did not have any NMS (mean age = 35.6 years), five had one NMS (mean age = 42.6 years), six had two NMS (mean age = 42.8 years), and three patients presented with three or more NMS (mean age = 59.3 years). REM sleep behavior disorder was reported by four patients. The only patient for which PSG results were available was patient #8; he presented cervical myoclonus in REM sleep which was considered as a variant of normality. Depressive symptoms were identified in five others, only one of whom was on antidepressants. Seven patients had a MoCa score < 26 (range, 19–23); these had a mean educational attainment of 5.0 ± 1.2 years, versus 10.5 ± 3.6 years in the group with MoCa ≥26.

**Table 2:** Scores of the scales used to evaluate non-motor symptoms of Parkinson’s disease in patients with Gaucher disease type 1 and comorbidities found (*n* = 23)

Patient	BDI	ESS	UMSARS	SST	MoCa	RBD-1Q	MDS-UPDRS III	Comorbidities
1	4	6	0	10	30	N	0	Hyperparathyroidism
**2**	16	11	1	10	26	N	0	Lactating, low vitamin B12 level
3	4	9	0	11	28	N	0	Ulcerative colitis (treated with aminosalicylate), low vitamin B12 level
4	5	8	1	12	29	N	0	None
**5**	3	17	0	11	23	Y	0	Smoking
6	4	9	1	11	26	N	0	None
7	3	11	1	10	26	N	0	Pulmonary hypertension
8	2	8	1	9	26	Y	0	None
9	12	11	1	12	27	N	0	High blood pressure (treated with angiotensin receptor blockers and diuretics), low vitamin B12 level
**10**	0	18	0	12	26	Y	0	Smoking
11	6	13	1	12	26	N	0	Asthma, irritable bowel syndrome (treated with antidepressants, spasmolytics, and a beta blocker)
12	7	3	1	12	30	N	0	Smoking; hypertension (treated with beta blocker)
13	2	10	0	10	29	N	0	None
14	4	15	0	12	29	N	0	Cardiomyopathy (treated with beta blockers)
**15**	25	1	2	10	22	N	0	Smoking, depression (treated with tricyclic antidepressants)
**16**	33	7	0	11	22	N	0	None
17	6	NP	0	12	26	N	0	Insomnia, anxiety (treated with nonbenzodiazepine hypnotics and selective serotonin reuptake inhibitor), low vitamin B12 level
**18** ^a^	14	12	0	6	26	N	0	Hypertension (treated with angiotensin-converting enzyme inhibitors).
**19** ^a^	17	4	2	4	22	N	0	Hypertension (treated with calcium channel blockers and beta blocker), osteoporosis, rhinitis
**20**	13	12	1	10	20	N	27	Hepatocellular carcinoma, hypertension (treated with calcium channel blockers and beta blocker)
21	3	7	0	11	26	N	7	Stroke at age 55; hypertension (treated with calcium channel blockers, angiotensin receptor blockers, and beta blocker)
**22**	4	13	2	9	19	Y	0	Obesity, osteoporosis, arrhythmia, low vitamin B12 level
**23**	16	3	1	10	23	N	0	Depression (treated with selective serotonin reuptake inhibitor), hypertension (treated with angiotensin-converting enzyme inhibitor)

Altered results are presented underlined. Bold type denotes patients who screened positive for more than one non-motor symptom of Parkinson’s disease*. BDI* Beck Depression Inventory (cutoff for depression > 14)*, ESS* Epworth Sleepiness Scale (cutoff for increased daytime sleepiness > 10), *UMSARS* Unified Multiple System Atrophy Rating Scale (cutoff for constipation ≥2), *SST* Sniffin’ Sticks Test (cutoff for hyposmia < 9/12), *MoCa* Montreal Cognitive assessment (cutoff for cognitive impairment < 26), *RBD-1Q* Single-Question Screen for REM Sleep Behavior Disorder (cutoff being a positive answer to the single question), *NP* not performed, *MDS-UPDRS III* motor symptoms of PD assessed with part III of the Unified Parkinson’s Disease Rating Scale. Low vitamin B12 level < 200 pg/mL. ^a^These patients underwent clinical examination and fiberoptic nasopharyngoscopy by an ENT doctor

A lower MoCa score was associated with greater GD severity as measured by the SSI (Zimran Severity Score Index, mean score 5.3 ± 3.6 in the MoCa < 26 group vs. 1.6 ± 1.7 in the MoCa ≥26 group, *p* = 0.016) and DS3 scores (mean score 2.7 ± 2.1 in the MoCa < 26 group vs. 0.7 ± 0.9 in the MoCa ≥26 group, *p* = 0.013), as well as with older age at diagnosis (mean age, 42 ± 17 years in the MoCa < 26 group vs. 24 ± 16 years in the MoCa ≥26 group, *p* = 0.028). Constipation was identified in three patients, and daytime sleepiness in 10 (only one patient in this group had a BDI score > 14). Neither daytime sleepiness nor constipation correlated significantly with any clinical or laboratory parameters (data not shown).

Two patients (#20 and #21) exhibited parkinsonian motor symptoms, and were also evaluated by a neurologist. Patient #21 had bradykinesia and loss of automatic movements, confirmed by the specialist, but no NMS. Patient #20 had bradykinesia and altered cognition and daytime sleepiness scores. However, both patients were on amlodipine, a calcium channel blocker that can jeopardize assessment of these motor symptoms, and will receive further evaluation.

The Sniffin’ Sticks smell identification test was abnormal in two patients (scores 6/12 and 4/12). Both clinical examination and fiberoptic nasopharyngoscopy were performed by an otorhinolaryngologist. Chronic sinusitis and atopic epithelium were identified on physical examination of patient #19, and neither patient complained of reduced smell perception.

## Discussion

In this study, it was found a high prevalence of NMS of PD among adult treated GD type 1 patients. The most common NMS was daytime sleepiness, followed by cognitive impairment.

In a series of five patients with concurrent GD and PD (GD-PD), the average age of PD onset was 53.8 years, and three patients presented with PD before the age of 50, which is earlier than what was found in other studies [14]. Some of our patients exhibited NMS, which could represent the beginning of the parkinsonian pathological process. There is no consensus as to whether the presence of these symptoms alone, especially when detected on cross-sectional evaluation, could indicate the start of a neurodegenerative disease. According to a 2-year follow-up study of GD patients and controls, many NMS worsened in the GD group at 2 years from baseline, demonstrating the importance of longitudinal follow-up [11].

Cognition was altered in 7 of 23 patients in our sample, and we found a negative correlation between MoCa < 26 and older age at diagnosis. We point out that milder forms of GD1 are expected to be diagnosed later, specially in developed countries with a high prevalence of N370S like Israel (N370S/N370S is considered to be a milder genotype). However, this is not the rule in Brazil: since the facilities for diagnosis of GD are not available countrywide, even the more severe patients are diagnosed later. Besides that, Rec alleles and L444P are highly prevalent in our cohort.

Low-normal range of vitamin B12 was already associated with PD and decreased cognition [15] but in our cohort only 1/7 patients with Moca < 26 had low vitamin B12 levels. Unfortunately, the biomarkers of functional vitamin B12 deficiency (methylmalonic acid and homocysteine levels) were not available for analysis.

GD-PD is characterized by a greater severity of cognitive deficits than in iPD [16]. In one study (*n* = 355) which compared patients with iPD, GD-PD, and PD with mutated *GBA1*, cognitive, motor, olfactory, and psychiatric symptoms were more severe in those with GD-PD and those with severe *GBA1* mutations than in those with iPD [17]. A study with the objective to characterize the cognitive profile of GD type 1 patients (*n* = 86) using computerized cognitive tests showed mild cognitive deficits when compared to healthy age-matched subjects [18]. In this study, older patients scored worse on these scores than younger patients and we also regard the finding that our patients with MoCa < 26 were older at diagnosis as a confounding factor.

Hyposmia was found in two of our patients, and both were referred to an ERT doctor for further assessment. In one of the patients, hyposmia was probably due to untreated chronic rhinitis. In a study with 84 participants (among patients with GD, controls and heterozygous for *GBA1* mutation) that evaluated NMS, hyposmia was considered the most early and sensitive prodromal marker of PD [6, 11]. There are no reports of hyposmia in patients with GD without PD, nor as a side effect of GD treatment [8]. Evaluation of the sense of smell, whether through a directed history-taking or through specific smell identification tests, is not performed routinely in clinical practice. We believe patients should be assessed for hyposmia more regularly, not only because smell identification allows better perception of taste and even identification of dangerous substances but also for its importance as a biomarker of PD.

The RBQ-1 for REM sleep disorder has a sensitivity and specificity of 92.2 and 87.7%, respectively [19]. One prospective cohort study performed in individuals with REM sleep behavior disorder showed that this prodromal criterion alone had 81.3% sensitivity and 67.9% specificity for conversion to PD/dementia with Lewy bodies at 4-year follow-up [20]. The BDI and UMSARS have also been validated to evaluate depression and constipation, respectively, and are widely used in clinical practice. In an Argentine cohort of 26 GD type 1 patients (mean age 22.3 ± 13.1, range 6–52 years), aiming at analysing the occurrence of prodromal markers of PD using questionnaires performed ad hoc, depression and constipation were found in three and two cases respectively, a rate similar to that of our sample [7]. No patient of the Argentinean cohort presented motor symptoms perhaps due to the fact that this cohort was younger than ours.

Regarding constipation, only 3 patients presented abnormal scores. However, one patient was also taking tricyclic antidepressant, a drug which is associated to this finding.

All patients in our cohort were tested for the 3′-UTR SNP (rs708606), which was associated with cognitive decline in a study of 426 patients with PD which compared mutation status with Brief Test of Attention scores [13]. Eight of our 23 patients were heterozygous for this SNP, with no significant association with clinical or laboratory parameters.

Two of 23 patients in our sample were also heterozygous for the complex allele L444P + E326K. There is evidence to consider the E326K variant as a risk factor for PD: it is significantly more frequent in PD patients compared to controls and has also been found to predict a more rapid progression of both cognitive dysfunction and motor symptoms in patients with PD when present [21, 22]. Neither carrier of this variant had any abnormalities on cognitive assessment in our study. As more and more studies are showing that *GBA1* variants influence heterogeneity in PD symptom progression, close follow-up is very important in this setting, especially for patients who harbor more than one pathogenic mutation.

Currently, there is no treatment that can stop the progression of PD. Diagnosis of this disease can represent a major burden to both patients and their families. There is little information in the literature about genetic counseling in populations at risk of PD; in one study of patients’ opinions concerning genetic counseling, 86.7% of the population at risk for PD believed that patients should be informed of this risk prior to screening for *GBA1* mutation carriers. Of these, 93.3% answered that prior knowledge of this risk would not have affected the decision to undergo screening [23].

The NMS of PD can represent a significant burden for patients. Even though there is not a disease modifying therapy for PD, some NMS can be managed symptomatically (e.g., constipation) and so the quality of life of the patients may be improved [24]. A comprehensive approach with a multidisciplinary team should be preferred, and patients should be referred to a specialist for evaluation whenever neurological complaints arise.

## Conclusions

The sensitivity and specificity of screening for NMS of PD varies widely, and there is not a single biomarker of PD that can predict outcomes. We believe that both patients with GD and heterozygous carriers of *GBA1* mutations should be aware of their increased risk for PD, and that patients over the age of 40 should be offered a multidisciplinary follow-up strategy aiming at an earlier diagnosis of PD. Such follow-up may include, for instance, non-invasive tests such as neurological examination and administration of multiple validated questionnaires. A follow-up study on this cohort is planned, which maybe will help us to elucidate more this complex disease interaction between GD and PD.

## Abbreviations

3′-UTR SNP (rs708606): single nucleotide polymorphism in the three-prime untranslated region (rs708606)
BDI: Beck Depression Inventory
ESS: Epworth Sleepiness Scale
GCase: Beta-glucocerebrosidase
GD: Gaucher disease
iPD: idiopathic Parkinson disease
MDS-UPDRS III: Part III of the Unified Parkinson’s Disease Rating Scale
MoCa: Montreal Cognitive assessment
NGS: Next-generation sequencing
NMS: Non-motor symptoms
PD: Parkinson’s disease
PSG: Polysomnography
RBD-1Q: Single-Question Screen for REM sleep behavior disorder
REM: Rapid eye movement
UMSARS: Unified Multiple System Atrophy Rating Scale
